# SAF: A Spectral-Adaptive Fusion Algorithm for Link Prediction in Complex Networks

**DOI:** 10.3390/e28070741

**Published:** 2026-07-01

**Authors:** Wen Liang, Chunyu Yang, Qiwei Liu, Wenbo Zhang, Hongliang Wang

**Affiliations:** 1 College of Information Science and Engineering, Shenyang Ligong University, Shenyang 110159, China; 15642633035@163.com (C.Y.); zhangwenbo@yeah.net (W.Z.); 2 Shenyang Institute of Computing Technology, Chinese Academy of Sciences, Shenyang 110168, China; wanghl@sict.ac.cn; 3 School of Public Administration and Policy, Dalian University of Technology, Dalian 116081, China; lqwwinfred@163.com; 4 University of Chinese Academy of Sciences, Beijing 100049, China; 5 Liaoning Province Human-Computer Interaction System Engineering Research Center Based on Digital Twin, Shenyang 110168, China

**Keywords:** complex networks, link prediction, spectral decomposition, Gaussian kernel

## Abstract

Accurate prediction of missing or potential links is crucial for understanding complex network dynamics and supporting applications such as social recommendation and infrastructure planning. To effectively exploit both global and local structural information, this study proposes a spectral-adaptive fusion (SAF) algorithm. SAF first constructs a spectral embedding matrix by retaining a subset of spectral components, from which a row-column normalized matrix and a Gaussian kernel matrix are derived. These matrices are then adaptively fused to produce link scores, using a common-neighbor-based mechanism that dynamically balances their contributions, capturing both local and global network features while mitigating the influence of highly central nodes. Energy retention and spectral gap analyses set the truncated ratio to 5%, resulting in an average runtime reduction of 71.0% across eight datasets. Under the AUC index, SAF achieves an average relative improvement of 2.22% over advanced graph neural network methods and 10.65% over matrix factorization approaches. Importantly, even at low training ratios, SAF maintains AUPR values above 0.91 on four networks and exhibits stable performance on recall, confirming its robustness and effectiveness for link prediction.

## 1. Introduction

Link prediction in complex networks is a fundamental topic in network science and plays a crucial role in uncovering the structural organization and evolutionary patterns of complex systems [1]. By inferring potential or missing links, link prediction helps reveal network robustness, information diffusion processes, and small-world properties, while also supporting interdisciplinary research across physics, computer science, and sociology [2]. Practically, link prediction has been widely applied in social media for user recommendation [3], in biological systems for identifying protein interactions and their functional modules [4], and in infrastructure or financial networks for optimizing resources and assessing risks [5]. Despite these advances, achieving accurate and stable link prediction in complex networks is still challenging due to the difficulty in balancing local and global structural influences during computation.

Existing link prediction methods can generally be grouped into five categories: similarity, maximum likelihood, matrix factorization, graph embedding, and graph neural network (GNN)-based approaches [6,7]. These methods exploit network topology, statistical dependencies, or representation learning capabilities to infer potential connections from different perspectives, and have been applied to various types of networks.

The essence of similarity-based methods lies in quantifying the structural similarity between node pairs under the assumption that nodes with higher similarity are more likely to establish links in the future [8]. Typically, these approaches employ different types of similarity indices: local measures rely on first-order or second-order neighborhoods, offering computational simplicity but limited global awareness, whereas global measures incorporate contributions from all possible paths, yielding a more comprehensive representation of network structure. Despite their efficiency and interpretability, they still struggle to simultaneously capture fine-grained local details and global topological patterns [9].

Maximum likelihood-based methods model the randomness and latent structural patterns of networks through probabilistic frameworks, estimating the likelihood of link formation via statistical inference [10]. These approaches emphasize interpretability by explicitly revealing the driving factors behind link formation, and can effectively handle noise, uncertainty, and missing data [11]. Typical models include hierarchical structure models, stochastic block models (SBMs), and exponential random graph models (ERGMs), with parameters fitted using maximum likelihood estimation or Bayesian inference to compute the conditional probability of links between node pairs [12]. For instance, SBMs partition nodes into groups and estimate inter-group connection probabilities, whereas ERGMs characterize link generation based on degree distributions and clustering coefficients [13]. Although such methods perform well in sociological and biological networks due to their statistical interpretability, their high computational cost limits scalability to large and dense networks.

Matrix factorization and spectral methods share a common foundation in characterizing the spectral properties of adjacency or Laplacian matrices [14]. Matrix factorization approaches, such as singular value decomposition (SVD) and nonnegative matrix factorization, aim to reconstruct the adjacency matrix through low-rank latent representations [15,16]. In contrast, spectral methods analyze the eigenvalues and eigenvectors of the graph Laplacian to capture global structural modes, effectively revealing community organization and latent correlations among nodes. Although mathematically related, spectral methods emphasize theoretical interpretability, whereas matrix factorization provides greater flexibility and scalability in large-scale networks. Due to the high computational cost of full eigendecomposition, recent studies have employed truncated spectra, randomized SVD, or polynomial filtering approximations to reduce complexity. Nevertheless, both paradigms still struggle to retain discriminative high-order structural information while maintaining computational efficiency.

Graph embedding methods typically employ unsupervised or semi-supervised learning, integrating network topology and potential semantic information, and are suitable for large-scale, heterogeneous, or dynamic networks [17]. To be specific, they involve two steps: first, generating node sequences via random walks to capture local and global structural information, to compress network topology into a low-dimensional space [18]; then, training embedding models through negative sampling or contrastive learning to ensure that node embeddings reflect network topological characteristics [19]. These methods often rely on large amounts of training data and can be sensitive to parameters, such as walk length, embedding dimension, and sampling strategy [20]. Additionally, while embeddings effectively compress structural information, they may obscure interpretable network properties, making it challenging to explain the learned representations.

GNN methods further extend this idea by leveraging neural architectures to capture complex topological dependencies and node attributes in an end-to-end manner [21]. Typical models include graph convolutional networks, graph attention networks, and temporal GNNs, all of which aggregate neighbor information to produce expressive node embeddings [22,23]. These methods often incorporate attention or temporal mechanisms to handle dynamic networks, and link probabilities are inferred through decoders or classifiers [24]. While GNN-based approaches have achieved impressive performance in social, biological, and transportation networks due to their ability to model spatiotemporal dependencies, they demand substantial computational resources. They are sensitive to the choice of hyperparameter settings and data quality.

Additionally, it is noteworthy that link prediction methods based on quantum walks have recently been proposed [25,26], and these studies suggest that quantum properties can address the challenge of balancing the contributions of local and global network features [27]. Quantum techniques achieve high accuracy in link prediction but rely heavily on eigendecomposition, which incurs high computational cost on the conventional von Neumann computing system [28]. Capturing global network patterns efficiently remains a major challenge in link prediction, as spectral and matrix factorization methods are often computationally expensive for large networks. Inspired by the connections between quantum walks and spectral approaches, the proposed spectral-adaptive fusion (SAF) algorithm selectively retains dominant spectral components through truncated decomposition, preserving the most informative global structures while integrating local neighborhood information via adaptive fusion, thereby achieving efficiency and accurate reconstruction of missing links. In this sense, SAF is designed to address several limitations of existing methods within a unified framework: local similarity indices often lack global structural awareness, full spectral or matrix-factorization methods incur high computational costs, and graph neural networks may reduce structural interpretability. The contributions of this study are summarized as follows.

- The SAF algorithm constructs a spectral embedding matrix by retaining a small subset of the spectral components and employs a quadratically constructed and row-column normalized matrix together with a Gaussian kernel matrix. An adaptive fusion mechanism based on common neighbors is designed to integrate these matrices for link scoring.
- Ablation studies demonstrate that this common-neighbor-based adaptive fusion effectively combines local and global structural information, significantly enhancing the link prediction performance of SAF.
- Guided by energy retention and spectral gap analyses, the truncated parameter of SAF is determined to 5%, leading to an average runtime reduction of 71.0% across eight datasets, confirming its computational efficiency.
- In terms of AUC, SAF outperforms advanced graph neural networks by 2.22% and matrix factorization methods by 10.65%, while maintaining high stability and predictive accuracy in terms of AUPR and recall indices even at low training ratios.

The remainder of this paper is organized as follows. Section 2 introduces the link prediction problem, eight representative baseline algorithms, and classical evaluation metrics. Section 3 proposes the SAF algorithm, detailing its spectral decomposition, quadratic similarity matrices, Gaussian kernel fusion, and the adaptive weighting mechanism. Section 4 evaluates link prediction performance on eight real-world networks, comparing results in terms of AUC, AUPR, and recall perspectives to demonstrate the superiority and robustness of SAF. Finally, Section 5 concludes the study and discusses future research directions.

## 2. Link Prediction, Baselines, and Estimated Indices

### 2.1. Definition of Link Prediction

**Figure 1:**
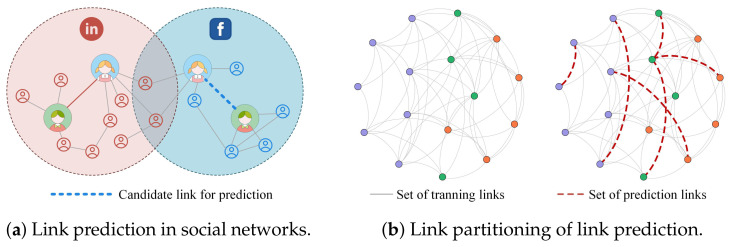
Application and visualization of link prediction.

In social networks, link prediction can be used to recommend potential friends by analyzing user interaction behaviors and historical connections, thereby enhancing user engagement on social platforms. Figure 1a presents a cross-platform user recommendation case study, which leverages mutual friendships to bridge LinkedIn and Facebook for potential connection suggestions. Given a certain undirected complex network ϱ=(V,E), where *V* represents the set of nodes and *E* denotes the set of links, |V|=N and |E|=M.

Assuming that set *E* in ϱ is randomly partitioned into two disjoint subsets $E^{T}$ and $E^{P}$ with a proportion η, where the set $E^{T}$ serves as training data and $E^{P}$ constitutes the missing (deleted) links to be predicted (also referred to as the prediction set). The two subsets obtained by partitioning satisfy $|E^{T}\left|=\eta |E\right|$ (η∈(0,1)), $E^{T}\cap E^{P}=⌀$, and $E^{T}\cup E^{P}=E$.

Assuming that the universal set $E^{U}$ refers to the set of all possible links in the network, including both existing and potential unobserved links. For the network $\tilde{G}=\left(V,E^{T}\right)$, the purpose of link prediction is to infer the links in $E^{P}$ by assigning likelihood scores to all possible links in the universal set $E^{U}$ using a predictive algorithm (model). Formally, the algorithm assigns scores to each link in $E^{U}$ to identify missing links. Figure 1b illustrates the partitioning of the link set into training and prediction sets. Therefore, when an algorithm or a model is employed for link prediction, it generates the similarity score for each link in $E^{U}$.

### 2.2. Baselines for Link Prediction

For link prediction, a variety of methods exist and are commonly used as baseline comparisons for the proposed SAF algorithm. These include local similarity indices, which emphasize neighborhood overlap; likelihood-based models such as the stochastic block model, which exploit community structure; and popular graph embedding and graph neural network approaches, which integrate node features and network topology. This section provides a comprehensive benchmark covering local, global, probabilistic, and representation learning.

#### 2.2.1. Resource Allocation

The Resource Allocation (RA) index is based on the principle of resource allocation, assuming that each common neighbor distributes a unit of resource evenly among its adjacent nodes [29]. This measure effectively suppresses the dominance of high-degree nodes and emphasizes pairs of low-degree nodes, showing strong robustness in sparse networks. The computational process of the RA algorithm can be expressed as:

(1) $$S_{ij}^{RA}=\sum_{w\in \Gamma (i)\cap \Gamma (j)}\frac{1}{k_{w}},$$

where Γ(i) and Γ(j) denote the neighbor sets of nodes *i* and *j*, respectively, and $k_{w}$ represents the degree of the common neighbor *w*.

#### 2.2.2. Adamic-Adar

The Adamic-Adar (AA) index penalizes highly connected neighbors logarithmically, further reducing their contribution to the similarity score [30]. It provides more precise discrimination in heterogeneous networks, such as social or collaborative systems, where degree disparity is significant. The similarity matrix obtained by AA can be defined as:

(2) $$S_{ij}^{AA}=\sum_{w\in \Gamma (i)\cap \Gamma (j)}\frac{1}{\log k_{w}}.$$

#### 2.2.3. Cosine Similarity

Cosine similarity quantifies the angle between adjacency vectors, capturing the overlap of local neighborhoods [31]. It is computationally efficient and requires no parameters, providing a straightforward measure of local structural resemblance.

(3) $$S_{ij}^{\cos}=\frac{A_{i}\cdot A_{j}}{{∥A_{i}∥}_{2}{∥A_{j}∥}_{2}},$$

where $A_{i}$ is the adjacency vector of node *i*.

#### 2.2.4. Stochastic Block Model

The Stochastic Block Model (SBM) first applies spectral clustering to partition nodes into clusters, then estimates inter-cluster connection probabilities to infer potential links [32]. It effectively captures mesoscopic structural patterns and is particularly suitable for networks with well-defined community structures. The similarity matrix using the SBM model can be defined as follows.

(4) $$S_{ij}^{SBM}=\theta_{z_{i}z_{j}},\;\theta_{ab}=\frac{E_{ab}}{N_{ab}},$$

where $z_{i}$ is the community label of node *i*, $E_{ab}$ denotes the number of existing edges between clusters *a* and *b*, and $N_{ab}$ is the total number of possible edges.

#### 2.2.5. Random Walk-Based Graph Embedding

Random walk-based embeddings (RWembed) refer to Node2Vec, a popular node embedding method widely used for link prediction [33]. Node2Vec generates biased random walks on the network to capture both homophily (nodes with similar neighbors) and structural equivalence (nodes with similar roles), producing node sequences that reflect local and global network structures. These sequences are then processed with a Skip-Gram model to learn low-dimensional embeddings. The probability of a link between nodes *i* and *j* is computed as

(5) $$P_{ij}=\sigma \left(h_{i}^{⊤}h_{j}\right),$$

where $h_{i}$ and $h_{j}$ are the node embeddings, and σ is the sigmoid function.

#### 2.2.6. DeepLink Framework

DeepLink [34] is a scalable, unsupervised framework that combines network structure and node content for link prediction. It generates customized paths considering edge weights, local neighborhoods, and community structure based on the Louvain method, learns structural embeddings using Word2Vec and content embeddings with Doc2Vec, and fuses them into unified node representations. A classifier then predicts missing links based on these features. DeepLink consistently outperforms baselines such as Node2Vec. The prediction function is simplistically defined as:

(6) $$\hat{y}\left(u,v\right)=\sigma \left(w^{⊤}\left(f_{u}⊙f_{v}\right)+b\right),$$

where $f_{u}=\left[S_{u};C_{u}\right]$ denotes the fused embedding of node *u*, ⊙ is the Hadamard product, and σ is the sigmoid function.

In short, these algorithms serve as robust baselines for evaluating SAF, striking a balance among interpretability, scalability, and predictive accuracy. Classical indices are efficient and intuitive, while embedding-based methods capture complex patterns and retain robustness in sparse or large-scale networks. These algorithms provide a benchmark to validate SAF’s effectiveness in various networks, demonstrating its advantages over existing approaches in terms of prediction accuracy and robustness.

#### 2.2.7. Neighborhood Overlap-Aware Graph Neural Networks

The Neighborhood Overlap-aware Graph Neural Networks (Neo) model addresses the limitation of conventional graph neural networks (GNNs) in link prediction [35], where structural information is often underutilized. Unlike standard GNNs, Neo first learns structural features for each node directly from the adjacency matrix and then computes link probabilities by emphasizing the overlap of neighborhoods. In the Neo model, the node structural features are generated as $x_{i}^{\mathrm{struct}}=F_{\theta }\left(A_{i}\right)$, where $F_{\theta }$ denotes a learnable function. Multi-hop neighborhood aggregation is performed using a weighted sum of adjacency powers, symbolically represented as $Z=g_{\Phi }\left(\sum_{l=1}^{L}\beta^{l-1}A^{l}X_{\mathrm{struct}}\right)$. Therefore, the final link prediction integrates structural and feature-based embeddings via a convex combination, i.e.,

(7) $${\hat{y}}_{ij}=\alpha \cdot \sigma \left(z_{i}^{T}z_{j}\right)+\left(1-\alpha \right)\cdot \sigma \left(s\left(h_{i},h_{j}\right)\right),$$

where α is a learnable parameter, $h_{i}$ is the feature-based GNN embedding, and σ denotes the activation function. This framework allows Neo to leverage both graph topology and node features efficiently, capturing multi-hop neighborhood overlaps that are crucial for accurate link prediction.

#### 2.2.8. Deep Autoencoder-like Non-Negative Matrix Factorization with $L_{2,1}$ Norm

The Deep Autoencoder-like Non-negative Matrix Factorization with $L_{2,1}$ Norm (NMF) model extends traditional non-negative matrix factorization approaches by incorporating a deep encoder-decoder architecture to capture hierarchical structures in networks and applying the $L_{2,1}$ norm to reduce random noise [36]. Assuming that *X* and $V_{p}$ represent the multi-layer decoder and the encoder maps, respectively, then the objective function jointly optimizes reconstruction and noise suppression, i.e.,

(8) $$\min_{W_{i},V_{i}}|X-W_{1}\ldots W_{p}V_{p}{|}_{F}^{2}+|V_{p}-W_{p}^{T}\ldots W_{1}^{T}X|F^{2}+\beta \left|V_{i}\right|2,1,\;W_{i},V_{i}\geq 0,$$

where β controls the strength of noise suppression. Pre-training is performed layer-wise to initialize the matrices, followed by alternating fine-tuning of $W_{i}$ and $V_{i}$ for the entire model. This approach effectively captures complex hierarchical dependencies between nodes while mitigating the influence of spurious links, providing robust and accurate link prediction across a variety of network structures.

### 2.3. Estimated Indices

To comprehensively assess the effectiveness of link prediction methods, three representative evaluation metrics are adopted: AUC (area under the receiver operating characteristic curve), AUPR (area under the precision-recall curve), and recall [31]. Table 1 summarizes the definitions and distinctions among these three evaluation indices for clarity and comparison.

AUC quantifies a model’s global ranking ability by measuring how well true links are prioritized over non-existent ones across all possible decision thresholds, with higher values indicating stronger discriminative power. In contrast, AUPR focuses on the balance between precision and recall, emphasizing the model’s robustness with limited training data and its ability to maintain high precision among top-ranked predictions, which is particularly important in sparse or imbalanced networks. Finally, recall assesses the practical utility of the model by computing the fraction of true links correctly predicted, reflecting its overall effectiveness in identifying existing connections.

## 3. Spectral Adaptive Fusion Algorithm for Link Prediction

**Figure 2:**
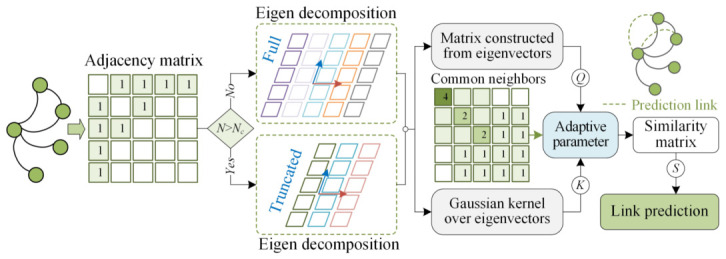
Illustration of the SAF algorithm.

To accurately identify missing links in networks, this study proposes the SAF algorithm. The algorithm begins by reconstructing the network through spectral decomposition to derive a row-column normalized spectral matrix, based on which node similarities are computed using a Gaussian kernel. By incorporating common neighbor information, an adaptive adjustment parameter is designed to balance and fuse the contributions of the normalized spectral matrix and the similarity matrix, thereby enabling link prediction in networks. Figure 2 schematically illustrates SAF’s core workflow, detailing the key computational stages from spectral decomposition to feature fusion.

### 3.1. Description of the SAF Algorithm

The graph $\tilde{G}$ is represented by its adjacency matrix $A\in R^{N\times N}$, each element in *A* can be defined as:

(9) $$A_{ij}=\left\{\begin{array}{cc} 1, & if(i,j)\in E,\\ 0, & otherwise \end{array}\right..$$

Matrix *A* encodes the presence or absence of links between nodes *i* and *j*, assuming *A* is unweighted and undirected.

The SAF algorithm employs spectral decomposition to extract structural representations for link prediction. To reduce the computational burden of full eigendecomposition, SAF retains only a subset of dominant spectral components for networks larger than a predefined threshold $N_{c}$, while using the full spectrum for smaller networks. Let *m* denote the number of selected spectral components, where m=N for $N\leq N_{c}$ and m=l for $N>N_{c}$. For the adjacency matrix *A*, denote the selected eigenpairs as $\left(\lambda_{r},u_{r}\right)$, r=1,…,m, where $u_{r}\in R^{N}$ is the eigenvector corresponding to eigenvalue $\lambda_{r}$. Accordingly, the spectral decomposition used in SAF can be formulated as:

(10) $$Au_{r}=\lambda_{r}u_{r},\;r=1,2,\ldots ,m,$$

Assuming that $U=\left[u_{1},u_{2},\ldots ,u_{m}\right]\in R^{N\times m}$ is the selected spectral embedding matrix, and $\Lambda =\mathrm{diag}\left(\lambda_{1},\lambda_{2},\ldots ,\lambda_{m}\right)\in R^{m\times m}$. Then, matrix *A* can be reconstructed by

(11) $$A\approx U\Lambda U^{T}.$$

when m=N, Equation (11) corresponds to the full eigendecomposition; when m=l<N, it gives the truncated spectral representation used by SAF.

Spectral features constitute the core representation for the SAF algorithm in restoring missing links within a network. As illustrated in Figure 2, SAF combines a row-column normalized spectral matrix *Q* with a Gaussian kernel matrix *K*, to integrate global spectral patterns and local higher-order neighborhood information. This initial spectral matrix *Q* is constructed from the squared selected eigenvectors and can be defined as:

(12) $$Q=\sum_{r=1}^{m}\left(u_{r}⊙u_{r}\right){\left(u_{r}⊙u_{r}\right)}^{T},$$

where $u_{r}$ denotes the *r*-th selected eigenvector of the adjacency matrix *A*, ⊙ represents element-wise multiplication, and *m* is the number of spectral components used in SAF. Directly using the initial matrix *Q* may bias the results toward nodes with larger spectral responses or higher centrality, while diminishing the contribution of peripheral nodes.

Accordingly, SAF applies a lightweight single-pass row-column normalization to the initial *Q*, improving score comparability and reducing central-node dominance without introducing additional iterative cost. Specifically, row normalization is first performed to balance the similarity scores assigned by each node:

(13) $${\tilde{Q}}_{ij}\leftarrow \frac{Q_{ij}}{\sum_{j}Q_{ij}}.$$

Column normalization is then applied to the row-normalized matrix to balance the accumulated similarity received by each node, reducing the overrepresentation of influential nodes while preserving their structural role:

(14) $$Q_{ij}=\frac{\tilde{Q}ij}{\sum_{i}{\tilde{Q}}_{ij}}.$$

Since the row-column normalized spectral matrix *Q* is constructed from the selected eigenvectors, it mainly captures global spectral structure and may not fully reflect local higher-order neighborhood variations. To address this, SAF applies a Gaussian kernel to the node embeddings obtained from the selected spectral matrix *U*. Let $z_{i}\in R^{m}$ denote the spectral embedding vector of node *i*, corresponding to the *i*-th row of *U*. The squared Euclidean distance between nodes *i* and *j* in the spectral embedding space is defined as

(15) $$D_{ij}^{2}={∥z_{i}-z_{j}∥}^{2}=\sum_{r=1}^{m}{\left(U_{ir}-U_{jr}\right)}^{2}.$$

The Gaussian kernel converts spectral distances into similarity scores via an exponential decay function, suppressing extreme values and improving the robustness of link prediction. The Gaussian kernel in SAF can be defined as follows:

(16) $$K_{ij}=\exp\left(-\frac{D_{ij}}{2\sigma^{2}}\right),$$

where $K_{ij}$ denotes the Gaussian-kernel similarity between nodes *i* and *j*, and σ is the kernel bandwidth. This transformation suppresses extreme distance effects and provides a nonlinear similarity measure that complements the global spectral matrix *Q*.

To balance the contributions of the spectral matrix *Q* and the Gaussian kernel matrix *K* for each candidate node pair, SAF introduces an adaptive parameter α, which adjusts their weights based on the neighborhood overlap between nodes. This design enables SAF to integrate global spectral features with local higher-order information. The final similarity matrix *S* is defined as:

(17) $$S_{ij}=\alpha_{ij}\cdot Q_{ij}+\left(1-\alpha_{ij}\right)\cdot K_{ij},$$

where α denotes the adaptive parameter, determined by the number of nodes and their common neighbors, and defined as:

(18) $$\alpha_{ij}=1-\frac{\log\left(C_{ij}+1\right)}{\log N}.$$

In Equation (18), $C_{ij}$ represents the common neighbor between nodes *i* and *j*, which can be computed from the square of the adjacency matrix, i.e.,

(19) $$C_{ij}={\left(A^{2}\right)}_{ij},\;\forall i\neq j,\;C_{ij}=0\;\;\mathrm{if}\;A_{ij}>0.$$

It is noteworthy that the objective of link prediction is to identify potential future links, not those already present in the network. Therefore, if a link exists between nodes *i* and *j*, the effect of their common neighbors is suppressed by setting $C_{ij}$ to 0 in Equation (19). This ensures that the adaptive parameter $\alpha_{ij}$ in SAF accurately reflects the neighborhood overlap of node pairs without existing edges, enabling a balanced weighting between the similarity matrices *Q* and *K*.

The adaptive parameter $\alpha_{ij}$ in Equation (17) balances the contributions of the similarity matrices *Q* and *K*, mitigating information loss from truncated spectral decomposition. Dominant eigencomponents in *Q* preserve global structural patterns but may omit finer local interactions. By incorporating the number of common neighbors between node pairs as an adaptive weighting factor, $\alpha_{ij}$ adjusts based on neighborhood overlap: a smaller overlap increases $\alpha_{ij}$, emphasizing global spectral features, while a larger overlap decreases $\alpha_{ij}$, giving more weight to *K* to capture local higher-order differences and nonlinear similarities. This strategy balances global and local information, improving link prediction performance under spectral truncation.

### 3.2. Implementation of SAF Algorithm

Algorithm 1 implements SAF for link prediction. As shown in lines 2∼8, the adjacency matrix is obtained, and spectral decomposition is performed, computing either the top-*l* eigenpairs for large networks or the full eigenpairs for small networks. In lines 9∼13, a spectral matrix *Q* is constructed and normalized. Local structural information is captured via the Gaussian kernel matrix *K* in lines 14∼16, while lines 17∼19 assign adaptive weights α based on common neighbors to compensate for information lost in spectral truncation partly. Finally, in lines 20∼23, matrices *Q* and *K* are fused using α, symmetrized, and zeroed on the diagonal to produce the final similarity matrix *S* for link prediction. The code for the SAF algorithm is publicly available at *https://github.com/bandit-wen/SAF_linkPrediction* (accessed on 10 October 2025).
**Algorithm 1** SAF Algorithm for Link Prediction**Require:** Network G=(V,E), truncated parameter *l*, threshold $N_{c}$; **Ensure:** Similarity matrix *S*;  1:A← adjacency matrix of *G*;  2:**if** $N>N_{c}$ **then**  3:     Compute the top-*l* eigenpairs $\left(\lambda_{r},u_{r}\right)$ of *A*;  4:     m←l;  5:**else**  6:     Compute all eigenpairs $\left(\lambda_{r},u_{r}\right)$ of *A*;  7:     m←N;  8:**end if**  9:$U\leftarrow [u_{1},u_{2},\ldots ,u_{m}]$;10:$U^{2}\leftarrow U⊙U$;11:$Q\leftarrow U^{2}{\left(U^{2}\right)}^{⊤}$;12:Normalize rows: $\tilde{Q_{ij}}\leftarrow Q_{ij}/\sum_{j}Q_{ij}$;13:Normalize columns: $Q_{ij}\leftarrow \tilde{Q_{ij}}/\sum_{i}\tilde{Q_{ij}}$;14:Let $z_{i}$ and $z_{j}$ denote the *i*-th and *j*-th rows of *U*;15:Compute $D_{ij}^{2}\leftarrow {∥z_{i}-z_{j}∥}^{2}$;16:$K_{ij}\leftarrow \exp\left(-D_{ij}^{2}/2\sigma^{2}\right)$;17:$C_{ij}\leftarrow A_{ij}^{2}$;18:Set $C_{ij}\leftarrow 0$ if edge (i,j) exists;19:$\alpha_{ij}\leftarrow 1-\log\left(C_{ij}+1\right)/\log N$;20:$S_{ij}\leftarrow \alpha_{ij}\cdot Q_{ij}+\left(1-\alpha_{ij}\right)\cdot K_{ij}$;21:Symmetrize *S*: $S\leftarrow (S+S^{⊤})/2$;22:Set diagonal items $S_{ii}=0$;23:**return** *S*;


### 3.3. Ablation Study of the SAF Algorithm

**Figure 3:**
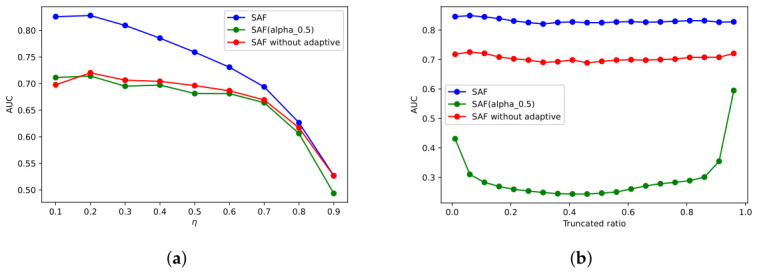
Ablation study of the SAF algorithm. (**a**) AUC performance under various test ratios. (**b**) AUC performance under various truncated ratios.

To evaluate the effect of the adaptive weighting mechanism in SAF, three ablation experiments were conducted on the same network under identical edge removal conditions. The experiments include: the original SAF algorithm, SAF with the α parameter fixed at 0.5, and SAF without the adaptive weighting mechanism. The experiments were performed on the email network, which contains 1133 nodes and 5451 edges. Figure 3 presents the AUC performance of the three configurations under varying edge deletion ratios.

As shown in Figure 3, the full SAF consistently outperforms both the fixed-α variants (SAF with α=0.5) and the non-adaptive variant (SAF without an adaptive weighting mechanism) across all deletion ratios. The performance drop is particularly noticeable under higher deletion rates for the ablated versions. These results indicate that the adaptive mechanism discussed in Equations (17)–(19) effectively integrates local structural information with global spectral features, enhancing the algorithm’s ability to identify missing links and demonstrating its objective effectiveness in link prediction.

### 3.4. Discussion of Spectral Truncation in SAF

The SAF algorithm leverages spectral truncation to efficiently capture the global structural patterns of a network while addressing the challenges of large-scale computation and incomplete local information.

(i) Structure Preservation in Spectral Truncation: Spectral truncation can be essentially viewed as a low-rank approximation technique aimed at reducing feature dimensionality while retaining dominant structural information. From the perspective of spectral graph theory, the leading eigenvectors of the adjacency matrix or its normalized variants correspond to the most representative structural patterns. In real-world networks, where the degree distribution follows a power law, spectral energy is highly concentrated in these leading components. Therefore, spectral truncation prioritizes dominant patterns, enabling substantial dimensionality reduction while preserving the essential structural features necessary for link prediction.

(ii) Adaptive Completion for Truncated Information: While spectral truncation preserves low-frequency dominant structures, it inevitably loses some high-frequency information, impairing the representation of fine-grained local structures. To address this issue, SAF algorithm introduces an adaptive fusion mechanism based on common neighbor features. The method dynamically modulates the fusion weights between the spectral feature matrices *Q* and *K* according to the number of shared neighbors, effectively integrating global structural information with local neighborhood characteristics and enhancing the truncated spectral representation.

(iii) Computational Complexity Advantage after Truncation: Full eigendecomposition typically requires $O(N^{3})$ computational complexity, which is impractical for large-scale networks. By computing only the top-*l* eigencomponents using the ARPACK-based truncated eigensolver in Python 3.12, SAF reduces the dimension-dependent spectral computation to $O(N^{2}l)$, while the actual eigensolver cost depends on matrix sparsity and convergence behavior. Spectral truncation thus serves as an effective feature compression technique, enabling SAF to exploit dominant global structural information with lower decomposition and spectral-similarity computation costs, while the dense pairwise scoring stage still requires $O(N^{2})$ memory.

Section 4 will determine suitable truncated parameters and analyzes SAF’s link prediction performance under spectral truncation.

## 4. Experimental Analysis of Link Prediction

This section optimizes the truncated parameters of the SAF algorithm and evaluate its link prediction performance using the AUC, AUPR, and recall metrics.

### 4.1. Experimental Setup

This study employs eight open-source complex networks from diverse domains, summarized in Table 2. These datasets can be categorized as follows. (i) social networks: Email, Hamsterster, and Moreno capture email exchanges, online friendships, and small-scale social interactions, respectively. (ii) co-authorship networks: NetSci, ca-GrQc, and CA-HepTh represent collaborations in network science, general relativity and quantum cosmology, and high energy physics, respectively; (iii) technological network: the p2p network from a peer-to-peer file sharing system. (iv) infrastructure network: the US Power Grid models the U.S. electrical transmission system. All datasets are publicly available from the Network Repository.

Table 2 summarizes the key topological characteristics of the network datasets used in this study. Here, *N* denotes the number of nodes, *M* the number of links, and $D_{MAX}$ the maximum node degree. The average degree 〈k〉 reflects the overall network connectivity, while the clustering coefficient *c* quantifies the local density of interconnections. The network diameter *d* indicates the longest shortest-path distance between any two nodes, and the assortativity coefficient ρ measures the tendency of nodes with similar degrees to connect.

In addition, this study uses the eight methods introduced in Section 2, including RA, AA, Cosine, SBM, RWembed, DeepLink, Neo, DANMFL, and NMF, as baselines to evaluate the link prediction accuracy of the proposed SAF algorithm across eight complex networks reported in Table 2.

### 4.2. Optimizing the Truncated Parameter in the SAF Algorithm

The spectral truncated parameter is critical to SAF performance and is determined using two metrics: the energy retention ratio and the spectral gap, both computed after sorting the eigenvalues in descending order by magnitude.

The energy retention ratio *E* measures the proportion of energy captured by the top-*l* eigenvalues relative to the total spectral energy. Since the contribution of each eigencomponent is determined by the squared eigenvalue, the eigenvalues are first sorted in descending order by magnitude, i.e., $\lambda_{1}\geq \lambda_{2}\geq \cdots \geq \lambda_{N}$. The energy retention ratio is then defined as

(20) $$E_{top-l}=\frac{\sum_{i=1}^{l}\lambda_{i}^{2}}{\sum_{i=1}^{N}\lambda_{i}^{2}}.$$

Generally, a higher proportion of retained spectral energy captures a greater amount of the network’s structural information [37].

The spectral gap measures the difference between consecutive eigenvalues sorted in descending order by magnitude. Typically, a pronounced spectral gap indicates a clear separation between dominant and subsequent spectral components, suggesting that the preceding eigenvalues play a more critical role in preserving the network’s structural information. The spectral gap between the *i*-th and (i+1)-th eigenvalues magnitudes is computed as:

(21) $$\Delta_{i}=|\lambda_{i}\left|-\right|\lambda_{i+1}|,\;i=1,2,\ldots ,N-1.$$

The spectral truncated parameter of the SAF algorithm is determined using Equations (20) and (21), selecting an optimal level that preserves the structurally significant eigenvalues for link prediction while filtering out noise.

The spectral truncated parameter is further determined using a maximum distance elbow method. Specifically, the starting and ending points of each curve are connected to form a reference chord, and the perpendicular distance from each candidate eigenvalue to this chord is calculated to quantify the degree of curve bending. For the curve constructed from the eigenvalue index and its corresponding measure, assuming that the starting and ending points are denoted as $P_{s}=\left(x_{s},y_{s}\right)$ and $P_{t}=\left(x_{t},y_{t}\right)$, respectively, then the perpendicular distance from any candidate point $P_{k}=\left(x_{k},y_{k}\right)$ to the chord $P_{s}P_{t}$ can be expressed as:

(22) $$d_{k}=\frac{\left|\left(x_{t}-x_{s}\right)\left(y_{s}-y_{k}\right)-\left(x_{s}-x_{k}\right)\left(y_{t}-y_{s}\right)\right|}{\sqrt{{\left(x_{t}-x_{s}\right)}^{2}+{\left(y_{t}-y_{s}\right)}^{2}}}.$$

Accordingly, the elbow point is defined as the index with the maximum perpendicular distance, i.e., $k^{*}=\arg\max d_{k}$.

**Figure 4:**
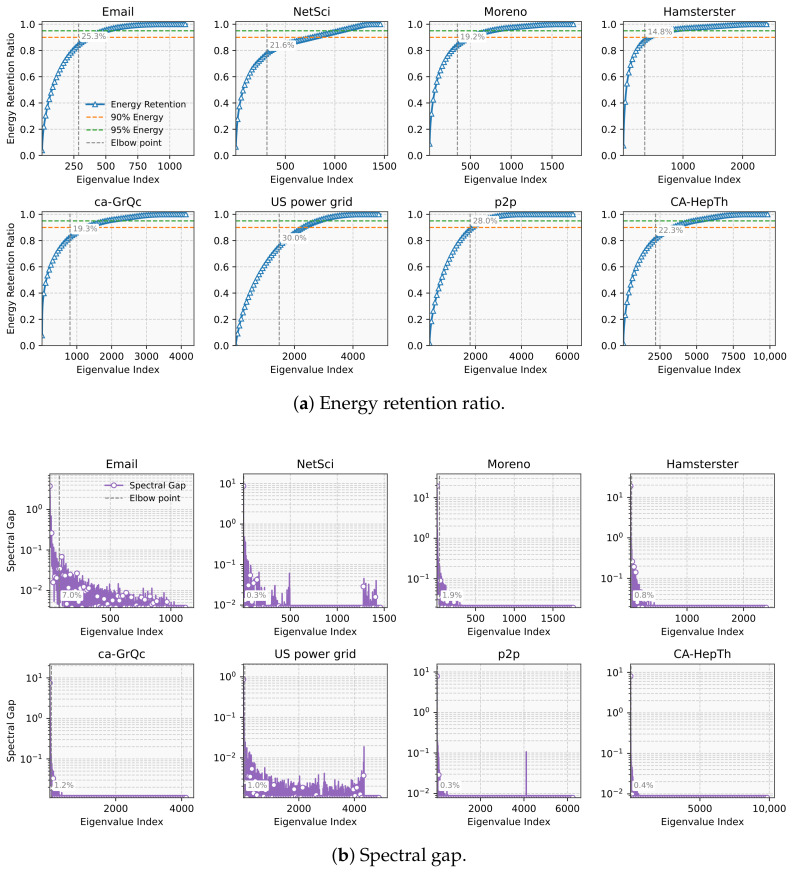
Spectral energy and spectral gaps across eight networks. In each subfigure, the gray dashed line marks the first elbow point. For clearer visualization, the y-axis in subplot (**b**) is shown on a logarithmic scale.

The results derived from Equations (20) and (21) are presented in Figure 4. In each subplot, the first elbow point, marked by a gray dashed line, separates the spectral components that capture dominant structural information from those that mainly contribute to local or microscopic variations.

As shown in Figure 4a, the cumulative energy retention curves rise rapidly at the early stage and then gradually flatten. At the first elbow points, retaining less than 30% of the spectral components is sufficient to preserve approximately 80% of the total spectral energy across the eight networks. This indicates that the main structural information is concentrated in a relatively limited subset of high-magnitude spectral components, while the remaining components mainly provide marginal cumulative contributions.

Further, Figure 4b identifies the first elbow points of the spectral gap curves within the same 30% spectral range. These elbow points appear much earlier than those in the energy retention curves, suggesting that the most pronounced separations among eigenvalue magnitudes are concentrated in only a small number of leading spectral components. Taken together, the two results indicate that a small group of high-magnitude eigenvalues captures the dominant global structure, whereas subsequent components mainly encode dispersed secondary or local variations. This provides empirical support for adopting a compact spectral truncation in SAF, rather than retaining a large proportion of the spectrum.

Based on the analyses for Figure 4a,b, the truncated parameter *l* for SAF is initially bounded by the average elbow proportions across the eight networks. Specifically, the upper bound is set using the mean elbow point from energy retention, calculated as (25.3+21.6+19.2+14.8+19.3+30.0+28.0+22.3)/8≈22.56%, while the lower bound is determined from the mean elbow point of the spectral gap experiment, i.e., (7.0+0.3+1.9+0.8+1.2+1.0+0.3+0.4)/8=1.61%.

**Figure 5:**
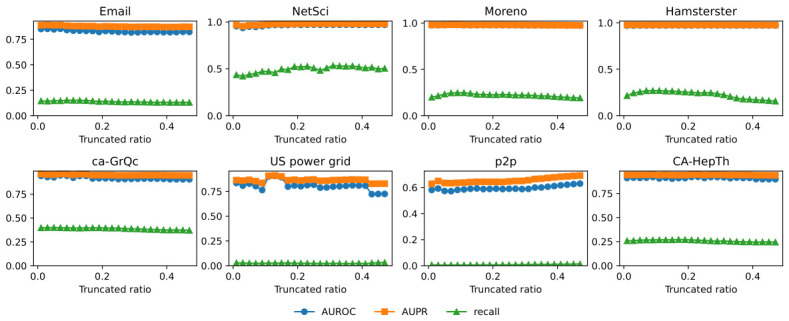
Variation of AUC, AUPR, and recall with various truncated ratios.

To further examine the relationship between truncated ratio and link prediction accuracy, SAF is evaluated under truncated ratios ranging from 1% to 50%, with a step size of 2% and the training parameter η set to 0.1. Figure 5 reports the corresponding AUC, AUPR, and recall results across the eight networks.

As shown in Figure 5, increasing the number of truncated eigenvalues does not lead to a significant improvement in prediction accuracy. For most networks, including Email, Moreno, Hamsterster, ca-GrQc, and CA-HepTh, AUC and AUPR remain relatively stable over a wide range of truncated ratios, indicating that a small subset of dominant spectral components is already sufficient to preserve the main structural information required for link prediction. This observation is consistent with the energy retention and spectral gap analyses in Figure 4, where the informative spectral components are concentrated in the leading part of the spectrum. Although some networks, such as p2p and US power grid, show local fluctuations or moderate changes with increasing truncated ratios, no general monotonic relationship can be observed between the number of retained eigenvalues and predictive performance.

The recall curves exhibit greater variability than AUC and AUPR, suggesting that the recovery of top-ranked missing links is more sensitive to network structure and truncated ratio. In particular, sparse networks such as US power grid and p2p show relatively low and stable recall values. This further suggests that retaining more spectral components does not necessarily improve the recovery of missing links in sparse or weakly locally closed networks.

According to Figure 4 and Figure 5, the candidate range for the truncation parameter can be determined as 1.61% to 22.56%. Within this candidate range, the predictive performance already stabilizes at low truncated ratios, while further increasing the number of retained spectral components does not yield consistent gains in AUC, AUPR, and recall. Accordingly, SAF adopts 5% as a compact setting that stays safely above the 1.61% lower bound while remaining far below the 22.56% upper bound, thus avoiding overly aggressive truncation and preserving dominant spectral information with lower computational cost.

**Figure 6:**
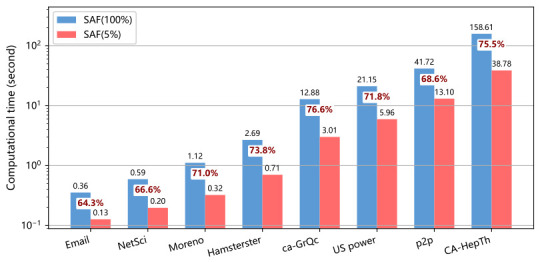
Time comparison between full and truncated decomposition for SAF.

With the truncated ratio set to 5%, the running time of the SAF algorithm is compared between full spectral decomposition and truncated decomposition that retains only the top 5% spectral components. All experiments are conducted on a unified hardware platform with the following specifications: 11th Core i5-11400H processor (2.70 GHz), 16.0 GB RAM, NVIDIA GeForce RTX 3050 Laptop GPU, running on Windows 11 operating system. The time comparison results are illustrated in Figure 6.

As depicted in Figure 6, when the truncated parameter is set to 5%, SAF reduces the running time by 64.3% to 76.6% compared with full spectral decomposition, with an average reduction of 71.0% across all eight network datasets. As the network scale increases, the efficiency advantage of spectral truncation becomes more evident, indicating that the proposed truncation strategy provides favorable scalability while maintaining predictive performance. Section 4.2, Section 4.3, Section 4.4 and Section 4.5 of this study will experimentally verify that the SAF algorithm maintains superior link prediction accuracy under the condition of retaining 5% spectral features.

### 4.3. Link Prediction Performance Under AUC Index

**Figure 7:**
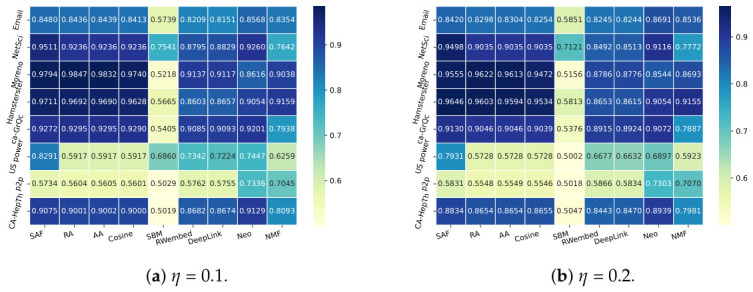
AUC performance using different methods at η=0.1 and η=0.2.

**Figure 8:**
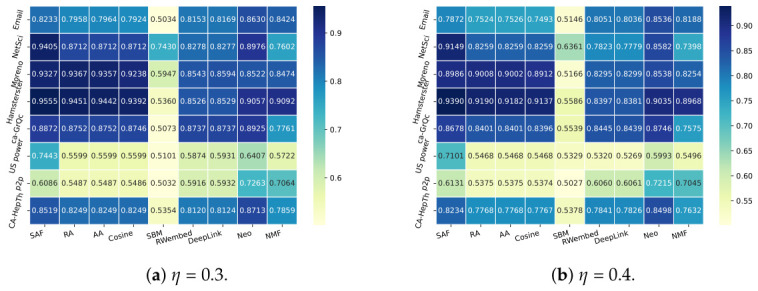
AUC performance of different methods under η=0.3 and η=0.4.

In the AUC index (refer to Section 2.3), a higher value indicates superior predictive capability. Figure 7 and Figure 8 report the AUC results of various methods across different training ratios, illustrating how algorithm performance evolves as the amount of observed data increases. The heatmaps employ color gradients to represent AUC values, where dark blue indicates higher predictive accuracy and light green corresponds to lower scores. Numerical annotations are also provided to explicitly display the AUC performance of each algorithm across all datasets.

As shown in Figure 7 and Figure 8, regardless of the training parameter η, the SAF algorithm consistently achieves the highest AUC on the NetSci, Hamsterster, and US Power Grid networks, demonstrating its significant advantage in link prediction. In particular, on the US Power Grid network, SAF substantially outperforms all baseline methods. Among traditional similarity-based approaches, RA and AA represent local similarity metrics, while Cosine represents a global similarity metric. SAF surpasses all of these, confirming that its adaptive mechanism effectively balances local and global features. Within graph embedding and graph learning methods, the Neo algorithm shows certain advantages, e.g., on the CA-HepTh network. However, overall, its performance remains less stable compared with SAF across most networks.

To more precisely assess the effectiveness of the SAF algorithm in link prediction, the conventional AUC metric is further refined by introducing a relative improvement indicator. This modification quantitatively measures the performance gains of SAF over baseline algorithms, providing a more objective benchmark for evaluating practical applicability. The average AUC is subsequently obtained by summing the AUC values across different training ratios $\eta_{k}$ (e.g., 0.1, 0.2, 0.3, and 0.4) and dividing by the total number of ratios, as formulated below:

(23) $$\bar{AUC}=\frac{1}{\phi }\sum_{k=1}^{\phi }AUC_{\eta_{k}},$$

where ϕ=4 represents the number of η values. This average quantifies the algorithm’s overall performance stability on a network. To further evaluate the relative improvement of SAF over the baselines, the improvement rate is defined as:

(24) $$RImp=1-\frac{{\bar{AUC}}_{\mathrm{baseline}}}{{\bar{AUC}}_{\mathrm{SAF}}}\times 100\%.$$

**Figure 9:**
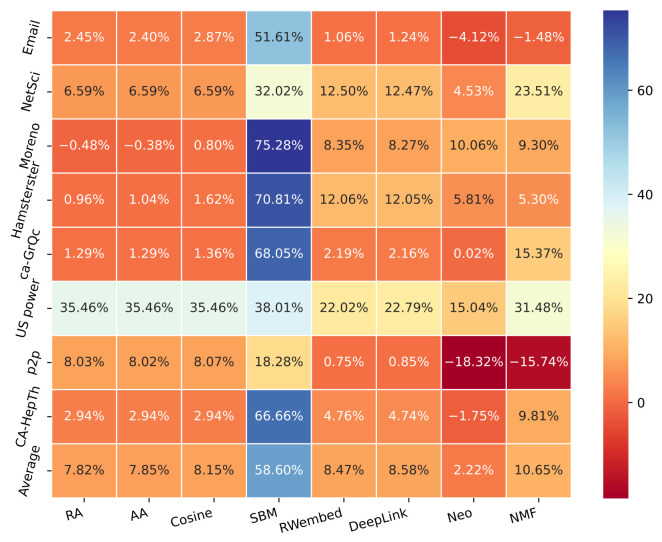
Relative AUC Improvement of the SAF Algorithm over Baselines.

This evaluation metric effectively highlights performance disparities among algorithms while maintaining fairness under varying noise levels. Based on Equations (23) and (24), the average AUC and relative AUC improvements of SAF and the eight baseline methods across the eight networks are presented in Figure 9.

As shown in Figure 9, SAF achieves positive average relative AUC improvements over all baseline methods across the eight networks. The gains are especially large compared with SBM, with an average improvement of 58.60%, indicating that a fixed block-structure assumption is insufficient to capture the heterogeneous link formation patterns in these networks. Compared with classical similarity indices, SAF also improves the average AUC by 7.82%, 7.85%, and 8.15% over RA, AA, and Cosine, respectively, suggesting that the fusion of spectral information and local neighborhood structure provides more discriminative link scores than relying on local overlap or vector similarity alone.

For representation learning and matrix-factorization-based methods, SAF obtains average improvements of 8.47%, 8.58%, and 10.65% over RWembed, DeepLink, and NMF, respectively. Although SAF does not outperform every baseline on every single network, the positive average gains against all methods show that it provides a more stable balance between global spectral structure and local neighborhood information. Compared with Neo, the average improvement is relatively smaller at 2.22%, but SAF still achieves competitive AUC performance without relying on deep neural architectures, further supporting its effectiveness and computational interpretability.

### 4.4. Link Prediction Under the AUPR Index

This section evaluates link prediction performance using the AUPR metric. As shown in Table 3, Table 4, Table 5 and Table 6, prediction becomes more challenging at lower training ratios, as the availability of observed edges decreases, leading to a general decline in algorithmic performance.

Table 3, Table 4, Table 5 and Table 6 report the AUPR results under different training ratios. Overall, SAF shows consistently strong performance across the four settings and achieves the best results in most networks. In particular, on NetSci, Moreno, Hamsterster, and ca-GrQc, SAF maintains high AUPR values regardless of the training ratio, with AUPR above 0.93 on NetSci, above 0.92 on Moreno, above 0.95 on Hamsterster, and above 0.91 on ca-GrQc. In such cases, SAF effectively exploits the retained high-magnitude spectral components to capture global structural patterns, while its adaptive weighting mechanism further strengthens local neighborhood signals.

The advantage of SAF is more evident on the US power grid network. Most baseline methods obtain AUPR values around 0.5∼0.6, whereas SAF remains above 0.74 under all training ratios. The US power grid has a very low clustering coefficient and a large diameter (as described in Table 2), indicating that local triangle-based similarity alone is insufficient to characterize potential links. Conventional indices such as RA, AA, and Cosine therefore show limited effectiveness. By contrast, SAF does not rely solely on local overlap; it uses spectral components to capture global connectivity patterns and then adaptively incorporates common-neighbor information.

Neo achieves the highest AUPR on the p2p network under all four training ratios. This may be related to the structural characteristics of p2p, which has the lowest clustering coefficient and relatively low assortativity among the datasets. In such a sparse technological network, local closure and degree-based homophily are weak, making common-neighbor signals less reliable. Neo’s neighborhood-overlap-aware neural aggregation is therefore better suited to capturing higher-order, non-triangular dependencies, explaining its advantage on the p2p network.

According to Table 3, Table 4, Table 5 and Table 6, the AUPR results show that SAF is especially effective when global spectral structure and local neighborhood information are both informative, and it remains robust even when local structural signals are weak.

### 4.5. Link Prediction Under the Recall Index

**Figure 10:**
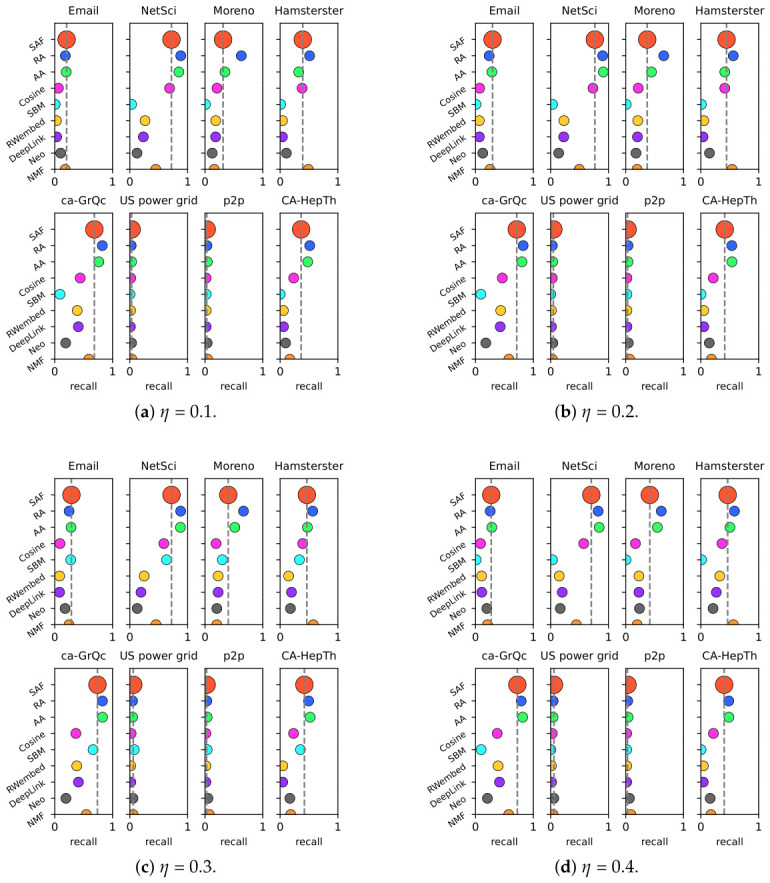
Link prediction performances under the recall index.

This section adopts the recall metric to evaluate all link prediction methods under different training ratios η. Recall index is computed based on the top-*k* ranked link scores, where *k* is set to the number of links to be predicted and therefore varies with the training ratio η. The corresponding experimental results are depicted in Figure 10, where different algorithms are distinguished by colored markers. A vertical dashed line is used to mark the recall value of SAF as a criterion.

As shown in Figure 10, SAF generally remains among the leading methods in terms of recall under different training ratios, indicating its strong ability to recover missing links among the top-ranked predictions. In the SAF algorithm, the truncated spectral components preserve the dominant global structure of the network, while the Gaussian kernel and the adaptive weighting mechanism further refine local link discrimination.

RA and AA also achieve competitive recall in NetSci, ca-GrQc, and CA-HepTH networks, which is expected because recall emphasizes whether true missing links can be retrieved among the top predictions. However, their performance depends heavily on local overlap and becomes less stable when such local signals are weak. NMF temporarily outperforms SAF on the Hamsterster network, suggesting that matrix factorization can be effective when the network contains strong low-rank or community regularities. Nevertheless, its recall varies more noticeably across networks, indicating weaker stability under heterogeneous structural conditions.

It is also notable that Neo is competitive in terms of AUC and AUPR, as shown in Section 4.3 and Section 4.4, but does not consistently achieve high recall. This suggests that neural neighborhood aggregation can improve overall ranking performance or the precision, but may not always place true missing links at the top of the prediction list. In contrast, SAF provides a more balanced mechanism: spectral truncation captures global structural patterns, while adaptive fusion with common-neighbor information enhances the retrieval of locally plausible missing links. These results indicate that SAF is effective not only in global ranking metrics but also in robustly recovering actual missing links under different training conditions.

In summary, the experimental results in Section 4 demonstrate that SAF achieves a favorable balance between computational efficiency and link prediction accuracy. Furthermore, SAF provides a robust and interpretable solution for link prediction across different complex networks.

## 5. Conclusions and Future Works

This study proposed a spectral-adaptive fusion (SAF) algorithm for link prediction in complex networks. The algorithm constructed a spectral embedding matrix by retaining a small portion of spectral components and integrated it with a Gaussian kernel method. Further, an adaptive weighting mechanism based on common neighbors was designed to combine local and global features, mitigating the overemphasis on highly central nodes. Guided by energy retention and spectral gap analyses, the truncated parameter was determined to 5%, which reduced the average runtime by 71.0% across eight networks. In terms of predictive accuracy, SAF achieved an average AUC improvement of 2.22% over advanced graph neural networks and 10.65% over matrix factorization approaches, while maintaining stable AUPR and recall performance even under limited training data.

Future work will focus on extending SAF to a wider range of network types to further enhance its applicability. One direction is its adaptation to directed networks, enabling the capture of asymmetric relationships inherent in citation, communication, and regulatory systems. Another avenue involves hypergraphs or higher-order networks, where SAF’s fusion of spectral and structural features could model multi-node interactions and preserve complex relational patterns. Additionally, the current study focuses on static networks; many real-world networks evolve, exhibiting temporal patterns and dynamic interactions. Extending SAF to handle temporal link prediction represents an important future direction. Potential approaches include incorporating time-aware spectral decomposition, dynamic Gaussian kernel mapping, or sequential embedding techniques to capture evolving structural and neighborhood patterns, enabling robust link prediction in dynamic network scenarios.

## Figures and Tables

**Table 1:** Comparisons of link prediction metrics.

Indices	Definitions	Differences Among Metrics
AUC	$\int_{0}^{1}\frac{\mathrm{TP}}{\mathrm{TP}+\mathrm{FN}}\left(\frac{\mathrm{FP}}{\mathrm{FP}+\mathrm{TN}}\right)\;d\frac{\mathrm{FP}}{\mathrm{FP}+\mathrm{TN}}$	Global link ranking performance
AUPR	$\int_{0}^{1}\frac{\mathrm{TP}}{\mathrm{TP}+\mathrm{FP}}\left(\frac{\mathrm{TP}}{\mathrm{TP}+\mathrm{FN}}\right)\;d\frac{\mathrm{TP}}{\mathrm{TP}+\mathrm{FN}}$	Robustness under limited training data
recall	$\frac{\mathrm{TP}}{\mathrm{TP}+\mathrm{FN}}$	Practical utility via correctly predicted links

TP: correctly predicting an existing link; FP: incorrectly predicting a non-existent link as existing; FN: missed prediction; TN: correctly identifying.

**Table 2:** Statistical characteristics of experimental complex networks.

Networks	*N*	*M*	$D_{\mathrm{MAX}}$	〈k〉	*c*	*d*	ρ
Email	1133	5451	71	9.6222	0.2201	8	0.0782
NetSci	1461	2742	34	3.7536	0.6936	17	0.4616
Moreno	1733	9131	364	10.3001	0.72081	8	−0.0488
Hamsterster	2426	16,630	273	13.7098	0.5375	10	0.0474
ca-GrQc	4158	13,421	81	6.4560	0.5568	17	0.6392
US power grid	4941	6594	19	2.6691	0.0801	46	0.0035
p2p	6301	20,777	97	6.5948	0.0108	9	0.0355
CA-HepTh	9877	25,998	65	5.2644	0.4714	18	0.2678

**Table 3:** AUPR performance using various methods with η= 0.1.

Networks	SAF	RA	AA	Cosine	SBM	RWembed	DeepLink	Neo	NMF
Email	**0.8877**	0.8406	0.8429	0.8400	0.5511	0.8534	0.8473	0.8756	0.8636
NetSci	**0.9644**	0.9236	0.9236	0.9236	0.6850	0.9296	0.9334	0.9250	0.7671
Moreno	0.9809	**0.9851**	0.9832	0.9684	0.5122	0.9217	0.9211	0.8714	0.9138
Hamsterster	**0.9770**	0.9701	0.9698	0.9603	0.5369	0.8741	0.8798	0.9106	0.9352
ca-GrQc	**0.9548**	0.9298	0.9299	0.9291	0.5319	0.9448	0.9452	0.9403	0.8161
US power	**0.8669**	0.5917	0.5917	0.5917	0.6474	0.8353	0.8299	0.7455	0.6273
p2p	0.6347	0.5566	0.5589	0.5470	0.5014	0.6301	0.6266	**0.7674**	0.7288
CA-HepTh	**0.9410**	0.9004	0.9004	0.9002	0.5012	0.9147	0.9141	0.9341	0.8369

Note: Bold values indicate the optimal AUPR score for each dataset.

**Table 4:** AUPR performance using various methods with η= 0.2.

Networks	SAF	RA	AA	Cosine	SBM	RWembed	DeepLink	Neo	NMF
Email	0.8836	0.8302	0.8322	0.8192	0.5543	0.8515	0.8510	**0.8886**	0.8798
NetSci	**0.9629**	0.9035	0.9035	0.9035	0.6408	0.9123	0.9169	0.9116	0.7795
Moreno	**0.9666**	0.9640	0.9626	0.9402	0.5086	0.8926	0.8922	0.8695	0.8857
Hamsterster	**0.9712**	0.9614	0.9599	0.9510	0.5455	0.8813	0.8794	0.9060	0.9324
ca-GrQc	**0.9442**	0.9051	0.9051	0.9040	0.5306	0.9338	0.9342	0.9277	0.8115
US power	**0.8372**	0.5728	0.5728	0.5728	0.5004	0.7806	0.7778	0.6902	0.5922
p2p	0.6374	0.5511	0.5528	0.5449	0.5009	0.6354	0.6311	**0.7617**	0.7301
CA-HepTh	**0.9236**	0.8655	0.8656	0.8657	0.5027	0.8956	0.8996	0.9175	0.8277

Note: Bold values indicate the optimal AUPR score for each dataset.

**Table 5:** AUPR performance using various methods with η= 0.3.

Networks	SAF	RA	AA	Cosine	SBM	RWembed	DeepLink	Neo	NMF
Email	0.8679	0.7979	0.7995	0.7880	0.5017	0.8421	0.8442	**0.8845**	0.8674
NetSci	**0.9554**	0.8712	0.8712	0.8712	0.6679	0.9015	0.9003	0.8980	0.7623
Moreno	**0.9500**	0.9390	0.9371	0.9155	0.5612	0.8759	0.8800	0.8661	0.8691
Hamsterster	**0.9643**	0.9464	0.9450	0.9370	0.5200	0.8666	0.8674	0.9065	0.9268
ca-GrQc	**0.9266**	0.8757	0.8758	0.8748	0.5041	0.9219	0.9217	0.9125	0.7942
US power	**0.7886**	0.5599	0.5599	0.5599	0.5062	0.7075	0.7117	0.6417	0.5732
p2p	0.6472	0.5454	0.5463	0.5408	0.5016	0.6260	0.6279	**0.7555**	0.7245
CA-HepTh	**0.8982**	0.8250	0.8251	0.8250	0.5191	0.8718	0.8737	0.8964	0.8133

Note: Bold values indicate the optimal AUPR score for each dataset.

**Table 6:** AUPR performance using various methods with η= 0.4.

Networks	SAF	RA	AA	Cosine	SBM	RWembed	DeepLink	Neo	NMF
Email	0.8331	0.7532	0.7537	0.7418	0.5079	0.8331	0.8311	**0.8757**	0.8410
NetSci	**0.9326**	0.8259	0.8259	0.8259	0.5863	0.8706	0.8674	0.8589	0.7427
Moreno	**0.9281**	0.9037	0.9024	0.8840	0.5088	0.8594	0.8589	0.8704	0.8459
Hamsterster	**0.9511**	0.9201	0.9187	0.9112	0.5324	0.8594	0.8559	0.9042	0.9152
ca-GrQc	**0.9117**	0.8406	0.8406	0.8396	0.5400	0.9024	0.9025	0.8927	0.7731
US power	**0.7413**	0.5468	0.5468	0.5468	0.5195	0.6433	0.6402	0.6007	0.5508
p2p	0.6418	0.5351	0.5355	0.5310	0.5014	0.6287	0.6278	**0.7469**	0.7179
CA-HepTh	0.8712	0.7812	0.7813	0.7811	0.5308	0.8532	0.8521	**0.8754**	0.7939

Note: Bold values indicate the optimal AUPR score for each dataset.

## Data Availability

All experimental networks shown in Table 2 are available for download at https://networkrepository.com/ (accessed on 10 October 2025), and the code for the SAF algorithm is publicly available at *https://github.com/bandit-wen/SAF_linkPrediction* (accessed on 10 October 2025).
